# Resilience of human gut microbiomes in autism spectrum disorder: measured using stiffness network analysis

**DOI:** 10.1128/spectrum.01078-24

**Published:** 2025-02-04

**Authors:** Hongju (Daisy) Chen, Bin Yi, Zhanshan (Sam) Ma

**Affiliations:** 1 School of Mathematics and Statistics, Guilin University of Technology, Guilin, China; 2 Computational Biology and Medical Ecology Lab, State Key Laboratory of Genetic Resources and Evolution, Kunming Institute of Zoology, Chinese Academy of Sciences, Kunming, China; Nanchang University, Nanchang, Jiangxi, China

**Keywords:** autism spectrum disorder (ASD), human gut microbiome, stiffness network analysis (SNA), potential microbial biomarkers

## Abstract

Autism spectrum disorder (ASD) affects an estimated 1%–2% of children worldwide, but its specific etiology remains unclear. In recent years, the gut microbiome’s role in ASD pathogenesis has garnered increasing attention. However, the exact relationship between microbiota and ASD—such as which microbial species significantly impact disease onset and progression—remains unresolved, and effective methods to measure microbial interactions are still lacking. In this study, we introduce an innovative stiffness network analysis (SNA) method to quantify changes in microbial network structure and identify disease-specific microbial bacteria theoretically. The SNA method was applied to reanalyze eight ASD gut microbiome data sets, encompassing 898 ASD samples and 467 healthy control (HC) samples from 16S-rRNA sequencing data. Key findings include the following: (i) an “allies” biomarker subgroup consisting of *Bacteroides plebeius*, *Sutterella*, *Lachnospira*, and *Prevotella copri* was identified; (ii) a profile monitoring score of 0.72 for the biomarker subgroup, indicating significant relationship changes between HC and ASD states, and (iii) a P/N ratio of biomarker subgroup in ASD-associated gut bacteria that was three times higher than that of HC microbiomes. Additionally, we discuss the non-monotonic relationship alterations within microbial sub-communities in the ASD gut microbiome.

**IMPORTANCE:**

It is crucial to assess alterations in network structure in different biological states in order to promote health. The stiffness network allows for the exploration of species interactions and the measurement of resilience in complex microbial networks. The objective of this study was to develop a stiffness network analysis (SNA) method for evaluating the contribution of microbial bacteria in differentiating disease samples from healthy control samples by examining changes in network stiffness parameters. Furthermore, the SNA method was employed on both simulated and real autism spectrum disorder gut microbiome data sets to identify potential microbial biomarker subgroups, with a particular focus on the relationship alterations within microbial networks.

## INTRODUCTION

The gut microbiome, often referred to as the “second genome,” plays a critical role in human health and disease (1, 2). Identifying gut microbial biomarkers for autism spectrum disorder (ASD) could prove valuable in improving clinical diagnosis and risk assessment for ASD patients (3–7). With the 16S-rRNA sequencing data, comparing the difference in individual microbial alteration (such as diversity or relative abundance) between the ASD and healthy control (HC) cohorts, the microbial biomarkers were detected based on differential abundance analysis of microbes and ecology co-occurrence networks (5–20).

Ecological networks offer a framework to explore selection mechanisms within microbial communities. In the selection process, the relative abundance of a species at a certain site is mainly determined by the fitness of the species to the environment. Hence, the interactions between species and environmental factors, as well as the interactions between species, will determine the selective force. Ecological networks are an effective tool for studying these interactions, helping in assessing the impact of disease on selective forces by identifying differences in microbial adaptation and interaction with environmental factors (21–23). The interactions between microbial species play a crucial role in shaping microbial community structure and dynamics, which in turn have a significant impact on disease, including ASD. In the human gut microbiome, these interactions are essential not only for maintaining microbial community stability but also for providing a stable environment for symbiotic organisms (3, 21, 22, 24, 25). Competition, cooperation, collaboration, and reciprocity among microbial species help stabilize the host’s gut microbial environment. For instance, ecological network analysis has shown that microbial composition and host health can be influenced by resource competition or the exchange of genetic material between species (24).

Several effective ecological network analysis tools have emerged in recent literature, including the MCODE algorithm (26), Core-Periphery Network Analysis (27, 28), High Salience Skeleton Network Analysis (29), and Network Module Structure Shift (23). These ecological network analysis tools not only provided ecological insights to the pathogenesis and individual risk assessment of microbiota-associated diseases but also enriched the basic theoretical research knowledge of diseases. For example, a dominance network analysis framework was used to explore potential resistance mechanisms to bacterial vaginosis (BV) microbiome dysregulation in “*non-lactobacillus-dominated*” women (21). *Finegoldia species* and s*taphylococcus epidermidis* were identified as the hubs of the skeleton network based on Core-Periphery Network Analysis, which play an important role in healthy *non-lactobacillus*-dominated vaginal microbiomes (21).

Variations in network topological structures can arise from different cutoff points selected across data sets, leading to the changes of different microbial interactions. To address this, Xiao et al. (23) introduced the Network Module Structure Shift model (Netmoss), which integrates multiple network data sets from different cohorts to identify biomarkers of microbiota-associated diseases by assessing shifts in microbial network modules. This approach allows for more robust detection of disease-related changes in microbial communities. The basic idea of Netmoss is to measure the importance of microbial species by assessing their role in transforming the network structure under different conditions. The Netmoss score is a metric, which can be considered as an indicator to measure the interindividual variation of human microbiome in the network structure framework under different states.

Relationship alterations (RA) of a defined sub-community with two or more microbes measure the changes (variance) of the sub-community under various biological conditions. The Profile Monitoring for Microbial Relationship Alteration (PM2RA) analysis method detects differences in the relative abundance of microbial species by estimating the distribution of Hotelling’s *T²* statistics (4). Hotelling’s *T^2^* statistics was used to project the relative abundance of two or more microbial taxa under different states into the same space; RA represents the difference in the distribution of *T^2^* statistics for individuals between two different states. The profile monitoring (PM) score is a scoring indicator of the PM2RA algorithm used to measure RA in each sub-community under different biological conditions. The range of PM score is generally between 0 and 1. If the PM score is 1, there is no overlap between the selected microbes under the two different conditions. If the PM score is 0, there is a complete overlap or similarity relationship between the selected microbes under the two different conditions. Higher PM scores indicated a more significant difference in *T²* statistics between the selected microbes under the two different conditions.

In the human gut microbiome, microbial species form an interconnected network through cooperative and competitive relationships. Permanent (sustaining) perturbations from outside may change the overall structure of the network, so it is no longer possible for the abundance of microbial species to depict the biogeography map of microorganisms, let alone the transition from health state to disease state. Thus, focusing solely on changes in individual microbial abundance, while neglecting the alteration of relationships between microbes, limits the effectiveness of ecological network analysis in identifying disease biomarkers. By accounting for these perturbations, constructing a method to measure the RA in microbial community co-occurrence networks between healthy control and disease states offers a deeper understanding of interspecies interactions within the human microbiome. This approach can enhance the identification of microbial biomarkers associated with disease progression.

Stiffness is a property expressing the level of consolidation of a structural framework, and thus, it is used to express the framework’s resistance against any cause inducing deformation and structural changes in structural engineering. Stiffness is a well-established concept in the field of structural engineering that is used within the context of structural analysis and designed to measure the level of deformation that sets of loads induce to structured elements. The measure of stiffness is also related to the ease with which an external force is propagated along the body of the structured framework, and it depends on the geometry (cutting area, length) and the composition (elasticity) of the elements (beams, columns) composing the framework.

In a conceptual analogy, Tsiotas (30) translated the stiffness concept from structural engineering to complex network analysis. This adaptation relies on three key mathematical elements, which provide the basis for using stiffness as a metric to evaluate the resilience of complex networks.

First, both a structured framework and a complex network enjoy discrete modeling into a graph composed of sets of nodes and edges (links). Particularly, in a structured framework, the structural elements (i.e., beams or columns) can be mapped into edges, and the edge intersections can be modeled into nodes. Similarly, a complex network is by default defined by a graph model composed of a set of nodes and links.

Second, in a complex network, edge weights (*w_ij_*) express the power of connectivity between nodes and thus configure the network’s capability to spread information along the network structure. Similarly, the elasticity moduli (*k_ij_*) of the structural elements (*ij*) define the capability of a structured framework to distribute the effect of external forces throughout the framework’s body, and thus, they configure the power of connectivity between nodes.

Third, the stiffness *k* of a structured framework is modeled into a square matrix *K_n×n_* (called the stiffness matrix or stiffness tensor), with the length equal to the number of nodes (intersections) (*n*) in the framework. The stiffness matrix includes the elastic moduli *K_n×n_* = [*k_ij_*] of the structural elements configured by nodes *i* and *j* and thus expresses a connectivity matrix *W_n×n_* = [*w_ij_*] of the framework’s graph model, in which edge weights are *w_ij_* = *k_ij_*.

The significance of stiffness networks includes two main points: (i) they lay a foundation for computational implementation as a metric for measuring resilience in microbial network structures. Microbial species interact, forming an interconnected network that can be modeled as a graph consisting of nodes (microbial species) and edges (relationships). The microbial stiffness network is derived from this structural framework, allowing for the development of a computational algorithm (stiffness network analysis [SNA] method) to capture various aspects of microbial stiffness, including stiffness scale, stability, and impact; (ii) in the human gut, studying stiffness networks of microbial species rather than individual species helps maintain community structure and provides a stable environment for symbiotic organisms. Evaluating RA in network structure across different biological states is crucial for improving health. Stiffness networks facilitate the exploration of interspecies relationships and measure the resilience of complex microbial networks.

In the context of ASD gut microbiome networks, such “forces” refer to structural changes within the ASD gut microbiome co-occurrence network, while “deformation” refers to alterations in the relative abundance of microbial bacteria between ASD and HC individuals. This study aimed to develop a SNA method to assess the role of microbial bacteria in distinguishing disease samples from healthy control samples by examining changes in network stiffness parameters. Additionally, we applied the SNA method to both simulated and real ASD gut microbiome data sets to identify potential microbial biomarker subgroups, focusing on the RAs within microbial networks.

## RESULTS

The gut microbiome network of HC and ASD patient was constructed; Table S2 lists the basic network properties of the HC and ASD cohorts. The ratio of positive (cooperative) links to negative (competitive) links (P/N ratio) serves as a measure of the balance of species interactions in the microbiome network, as noted by Ma (31). Our study found P/N ratios of 3.567 for HC and 26.417 for ASD, indicating that the ASD patients’ microbial species are predominantly cooperative.

In this study, we hypothesized that the observed differences (displacement) in the relative abundance of gut microbial species between ASD and HC cohorts are driven by changes (cause) in the average weight of the connections (edges) in the microbial network structure. To identify potential microbial biomarkers associated with ASD, we applied the SNA method, focusing on changes in network stiffness parameters between the HC and ASD cohorts. This approach allowed us to examine the structural differences in the gut microbiome network of individuals with ASD.

### Quantifying network stiffness parameters of ASD gut microbiome with the SNA method

To quantify the changes (variance) in network stiffness parameters between HC and ASD patients, the SNA method was applied to eight ASD gut microbiome data sets. Table 1 presents the SNA results. Key findings include the following:

(i) In approximately 25% (2/8) of the data sets, the mean species stability was less than 0.5, while 75% (6/8) exceeded this threshold. This indicates that in most ASD gut microbiome data sets (75% or 6/8), interspecies relationships are relatively stable.

**TABLE 1:** Mean (minimum and maximum) stiffness parameters of the Species Co-occurrence Network in the gut microbiome of patients with ASD^*a*^

Date sets	Force (*f*)	Displacement (*d*)	Stiffness scale (*s*)	Impact (*I*)	Stability (*S*)
D1	0.007 [0, 0.028]	57.681 [0.096, 397.645]	0 [0, 0.008]	6.217 [1.001, 297.864]	0.631 [0.004, 0.999]
D2	0.026 [0, 0.114]	2246.4 [0.167, 112546.26]	0 [0, 0]	78.1 [1.012, 2225.254]	0.209 [0.001, 0.994]
D3	0.003 [0, 0.017]	84.778 [0.006, 1419.179]	0.001 [0, 0.01]	5.809 [1, 376.268]	0.61 [0.005, 1]
D4	0.006 [0, 0.063]	203.632 [0.08, 2649.32]	0 [0, 0.005]	3.882 [1.001, 50.841]	0.663 [0.01, 0.998]
D5	0.137 [0, 0.381]	129.185 [0.01, 2532.041]	0 [0, 0.004]	183.257 [1.001, 5348.2]	0.432 [0, 0.998]
D6	0.004 [0, 0.025]	90.293 [0.001, 1698.617]	0 [0, 0.004]	2.81 [1, 59.512]	0.702 [0.019, 0.999]
D7	0.008 [0, 0.042]	216.986 [0.027, 4227.344]	0 [0, 0.006]	54.889 [1, 4981.545]	0.51 [0, 1]
D8	0.008 [0, 0.054]	62.038 [0.028, 1096.988]	0 [0, 0.006]	9.073 [1, 424.14]	0.608 [0.006, 0.999]

^*a*^ *D*_*i*_, the data set number (e.g., D1 for data set 1); *f*, the force parameter within the stiffness network structure; *d*, the displacement parameter in the stiffness network structure; s, the stiffness scale parameter in the stiffness network structure; *I*, the impact parameter in the stiffness network structure; *S*, the stability parameter in the stiffness network structure.

(ii) The average stability of individual microbial species in ASD gut microbiomes was 0.550, with values ranging from 0.209 to 0.702. This suggests that that the average strength of the response (disturbance or interference) of the nearest neighbor species to individual specie was relatively lower. In other words, individual microbial species in the ASD gut microbiome are less susceptible to disruption from neighboring species, potentially reflecting a tendency toward cooperation and collaboration within the microbiome.

### Identifying microbial biomarkers of ASD gut microbiome using the SNA method

To identify microbial biomarkers based on changes in network stiffness parameters, we conducted 1,000 random permutations for each data set and applied the SNA method to the random networks. Further details about the random permutation algorithm can be found in the “Materials and Methods” section.

By comparing the changes in network stiffness parameters of microbial species between real and random network structures, the ASD gut microbial bacteria were classified into significantly increased or significantly decreased groups (at both species and genus levels). The significantly increased (or decreased) bacteria are sensitive to interference during the development and occurrence of ASD patients, and their network stiffness parameters in random network structure are significantly higher (or lower) than in real network structure, as shown in Table S3.

Table 2 summarizes the proportion of sensitive microbial bacteria in the ASD gut microbiome based on three stiffness network parameters: stiffness scale (*s*), impact (*I*), and stability (*S*). The results indicate that the proportion of microbial bacteria showing significant changes in stiffness scale was relatively low (2.5% = 0.762% + 1.7%). However, the proportion of bacteria exhibiting significant differences in the impact and stability parameters of the ASD gut microbiome network was approximately 26% and 37%, respectively. These findings suggest that, across the eight ASD gut microbiome data sets, about 30% of microbial bacteria exhibits significant differences in network stiffness parameters compared with the HC cohort.

**TABLE 2:** Proportion of significant species in gut microbiome of patients with ASD identified by SNA method^*a*^

Date sets	Stiffness scale (*s*)			Impact (*I*)			Stability (*S*)			Numbers of species^*b*^
Significant difference	*Sig*		*No Sig*	*Sig*		*No Sig*	*Sig*		*No Sig*	*–*
	*Sig. in*	*Sig. de*		*Sig. in*	*Sig. de*		*Sig. in*	*Sig. de*		
**Class**	***R_i_*< *R***	***R_i_* > *R***	***R_i_* = *R***	***R_i_* < *R***	***R_i_* > *R***	***R_i_* = *R***	***R_i_* < *R***	***R_i_* > *R***	***R_i_* = *R***	–
D1	0%	0%	100%	0%	30.6%	69.4%	25.9%	0%	74.1%	85
	0/85	0/85	85/85	0/85	26/85	59/85	22/85	0/85	63/85	
D2	1.1%	0.5%	98.4%	0%	3.2%	96.8%	13.2%	0%	86.8%	190
	2/190	1/190	187/190	0/190	6/190	184/190	25/190	0/190	165/190	
D3	1.5%	1%	97.4%	0%	36.1%	63.9%	45.4%	0%	54.6%	194
	3/194	2/194	189/194	0/194	70/194	124/194	88/194	0/194	106/194	
D4	0.8%	2.5%	96.6%	0%	58.8%	41.2%	57.1%	0%	42.9%	119
	1/119	3/119	115/119	0/119	70/119	49/119	68/119	0/119	51/119	
D5	0%	2.9%	97.1%	3.9%	7.8%	88.3%	74.8%	0%	25.2%	103
	0/103	3/103	100/103	4/103	8/103	91/103	77/103	0/103	26/103	
D6	0.8%	0.8%	98.4%	0%	30.9%	69.1%	31.7%	0%	68.3%	123
	1/123	1/123	121/123	0/123	38/123	85/123	39/123	0/123	84/123	
D7	0%	4%	96%	0%	13.4%	86.6%	22.1%	0%	77.9%	149
	0/149	6/149	143/149	0/149	20/149	129/149	33/149	0/149	116/149	
D8	1.9%	1.9%	96.2%	0%	21.8%	78.2%	27.6%	0%	72.4%	156
	3/156	3/156	150/156	0/156	34/156	122/156	43/156	0/156	113/156	
**Mean (%)**	**0.762**	**1.7**	**97.513**	**0.488**	**25.325**	**74.188**	**37.225**	**0**	**62.775**	**100**
**Std. err.**	**0.257**	**0.483**	**0.487**	**0.487**	**6.334**	**6.158**	**7.235**	**0**	**7.235**	**0**

^*a*^ *Sig*, significant difference; *No Sig*, no significant difference; *Sig in*, species with significantly increased stiffness parameter; *Sig de*, species with significantly decreased stiffness parameter; *D*_*i*_, the data set number (e.g., D1 for data set 1); *R*_*i*_, the value of the stiffness parameter in the random network; *R*, the value of the stiffness parameter in the real network; *R*_*i*_ < *R* represents the proportion (*proportion* = number of species with stiffness measured levels/total number of species) of the value of the stiffness parameter in the random network that is lower than the corresponding value in the real network; Mean (%) denotes the mean value; Std.err signifies the standard error.

^*b*^ "-", indicates that such a value does not exist.

### Identifying microbial biomarkers of ASD gut microbiome using other methods

To evaluate the performance of the SNA method in inferring network RAs (i.e., distinguishing ASD disease samples from HC samples), we compared it with two other algorithms: PM2RA algorithm and Netmoss algorithm. These methods were introduced to measure the changes (variance) in gut microbial relationships in patients with ASD.

The PM2RA method was applied to quantify the alterations in interspecific relationships between the healthy control and ASD disease states. Microbial biomarkers were identified as “drivers” of this transition based on PM scores (FDR < 0.05 and PM score > 0.6), which reflect significant RAs among microbial modules.

These disease-specific microbial bacteria were identified as biomarkers based on Netmoss score (*Netmoss* > 0.6), implying significant changes in interspecific relationships from HC to ASD. In this context, species with higher Netmoss scores suggest that these microbial interactions are key drivers in the transition from health to ASD disease. To avoid potential bias from different microbiota data sets, we only applied the Netmoss analysis method to identify biomarkers for each case study data set, without involving the integration of multiple data sets.

By applying three analysis methods (SNA, PM2RA, and Netmoss), we identified several novel or previously reported gut microbes implicated in ASD disease, and the union sets of the separate data sets of the same format were further exhibited in Tables S3 and S4 and Table S5, respectively.

### Evaluating the performance of the SNA method in inferring network RAs

To assess the effectiveness of the SNA method on ASD gut microbiome data sets, we began by randomly selecting 50% of the samples from each data set to identify microbial bacteria associated with ASD. The remaining 50% of the samples was used as a validation data set (D9). This process was repeated 1,000 times for each data set to ensure robustness.

**Fig 1:**
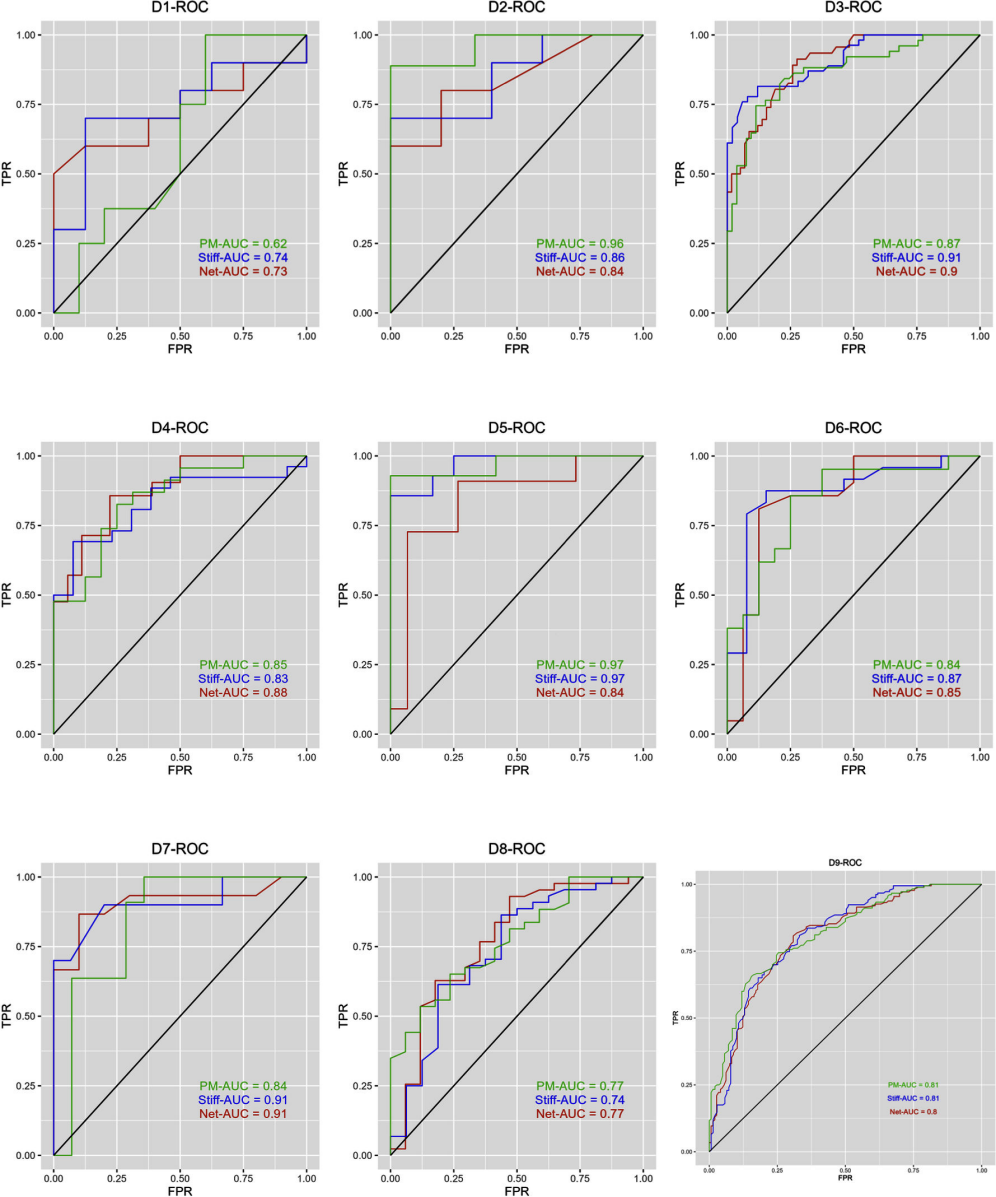
RF model of SNA method for distinguishing ASD disease and HC samples. FPR, false-positive fraction; TPR, true-positive fraction; PM-AUC, area under ROC curve predicted by PM2RA framework; Stiff-AUC, area under ROC curve predicted by SNA method; Net-AUC, area under ROC curve predicted by Netmoss modeling. The closer the AUC value to 1, the more accurate the classification effect is.

We further predict taxonomic and functional microbiome signatures of biomarkers that distinguish ASD disease samples from HC samples; random forest (RF) models were applied to the eight ASD gut microbiome data sets and an independent validation data set (D9). The RF models included microbial biomarkers detected by the SNA method (RF-Stiff), the PM2RA method (RF-PM), and the Netmoss method (RF-Net) (see Fig. 1). From the results in Fig. 1, we can summarize the following key findings:

(i) RF-Stiff model performance: the RF-Stiff model consistently exhibited higher average area under the curve (AUC) values on the receiver operating characteristic curve (ROC) across eight data sets. This suggests that the predictive microbiome signatures identified using the SNA method demonstrated high accuracy in the validation data sets.

(ii) AUC range for RF-Stiff model: the average AUC values for the RF-Stiff model ranged between 82% and 95% across the nine different validation data sets, indicating the strong predictive effect of the SNA-identified microbial biomarkers.

(iii) Robustness in independent validation: the RF models were applied to an independently validated data set, D9, where the AUC values on the ROC curves for the three algorithms were approximately 0.81. This suggests that variations in network structure might be an effective strategy to distinguish disease-specific microbial bacteria and merit further investigation.

These findings underscore the robustness of the SNA method in identifying microbial biomarkers for ASD and its potential as a reliable tool for gut microbiome analysis.

### Mining biomarker subgroups of ASD gut microbiome

To further investigate the interactions between biomarkers and other symbiotic microbial species in ASD patients, a network module method was applied to investigate biomarker subgroups across nine ASD gut microbiome data sets.

**Fig 2:**
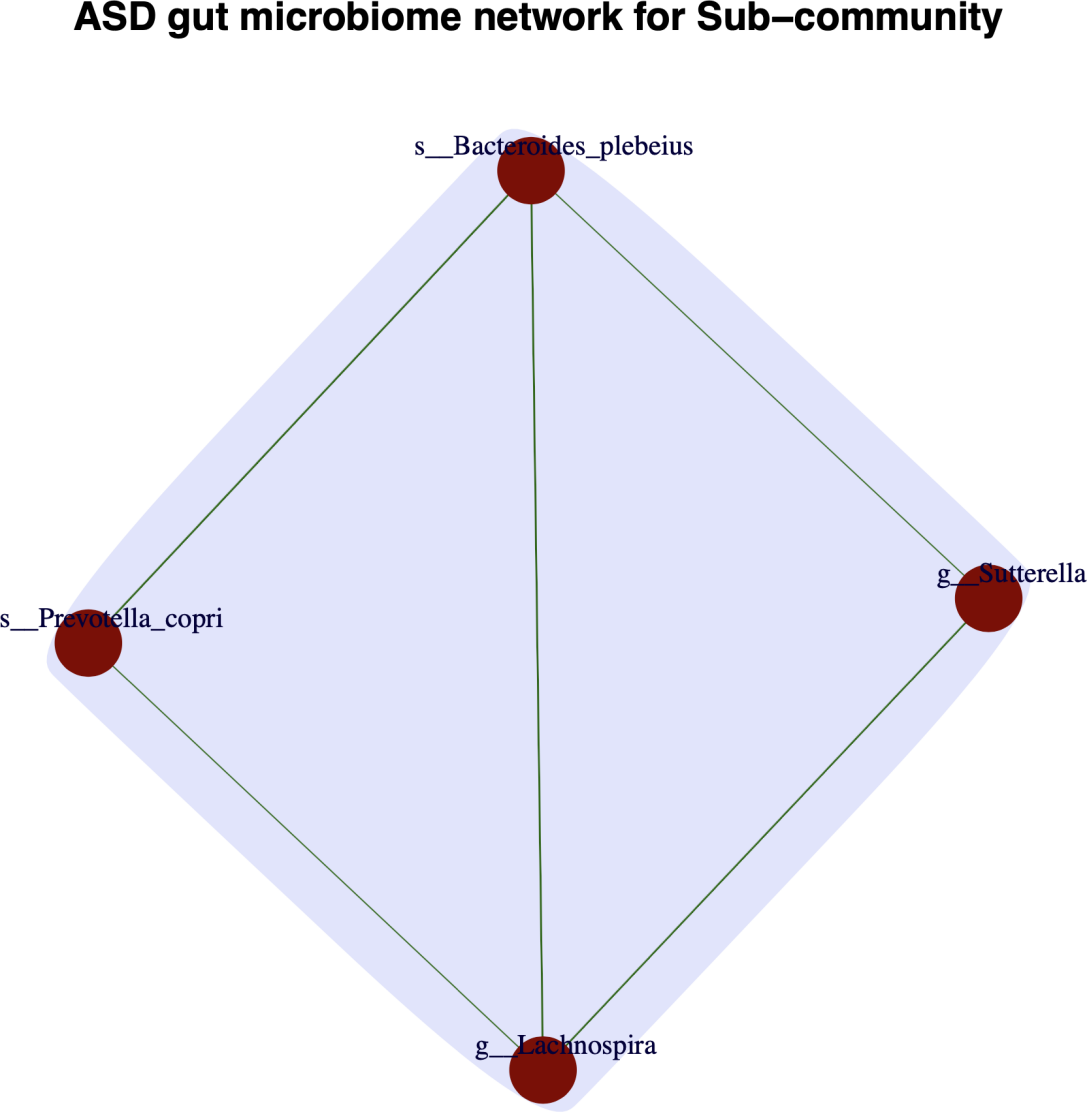
An “allies” biomarker subgroup consisting of four biomarker bacteria in the gut microbiome of patients with ASD (edge in green is a positive correlation between two-microbial bacteria)

At both the species and genus levels, *Bacteroides plebeius*, *Sutterella*, *Lachnospira*, and *Prevotella copri* formed a biomarker subgroup, displaying a positive correlation, as shown in Fig. 2. Notably, *Bacteroides plebeius* and *Sutterella* were identified as enriched bacteria in both the ASD and HC gut microbiomes using the specificity diversity (SD) concept and the specificity diversity permutation (SDP) test (32). *Prevotella copri*, however, was not significantly enriched in either ASD patients or HC cohorts (32). It is interesting that the *Prevotella copri* formed a triangular positive correlation with the other enriched species (*Bacteroides plebeius* and *Lachnospira*). The other three microbial bacteria (*Bacteroides plebeius*, *Sutterella*, and *Lachnospira*) also showed a triangular positive correlation. These results demonstrated a cooperative relationship between these four bacteria.

The PM score of the PM2RA framework, ranging from 0 to 1, was 0.72 (FDR < 0.05, *P* < 0.001, Fig. S1), which assesses the RA of the biomarker subgroup at two different conditions. Although the relative abundance of these four bacteria did not differ significantly between HC and ASD cohorts (Fig. S2), the PM score indicated a considerable RA within the subgroup (PM score = 0.72), with statistically significant differences (*P* < 0.001). These results suggest that the transformation in Hotelling’s *T*^2^ statistics for the microbial subgroup not only is a key signature distinguishing healthy control samples from ASD samples but also provides deeper insight into the pathogenesis and etiology of ASD.

### Constructing and analyzing the first-order neighbor network of microbial biomarkers

**Fig 3:**
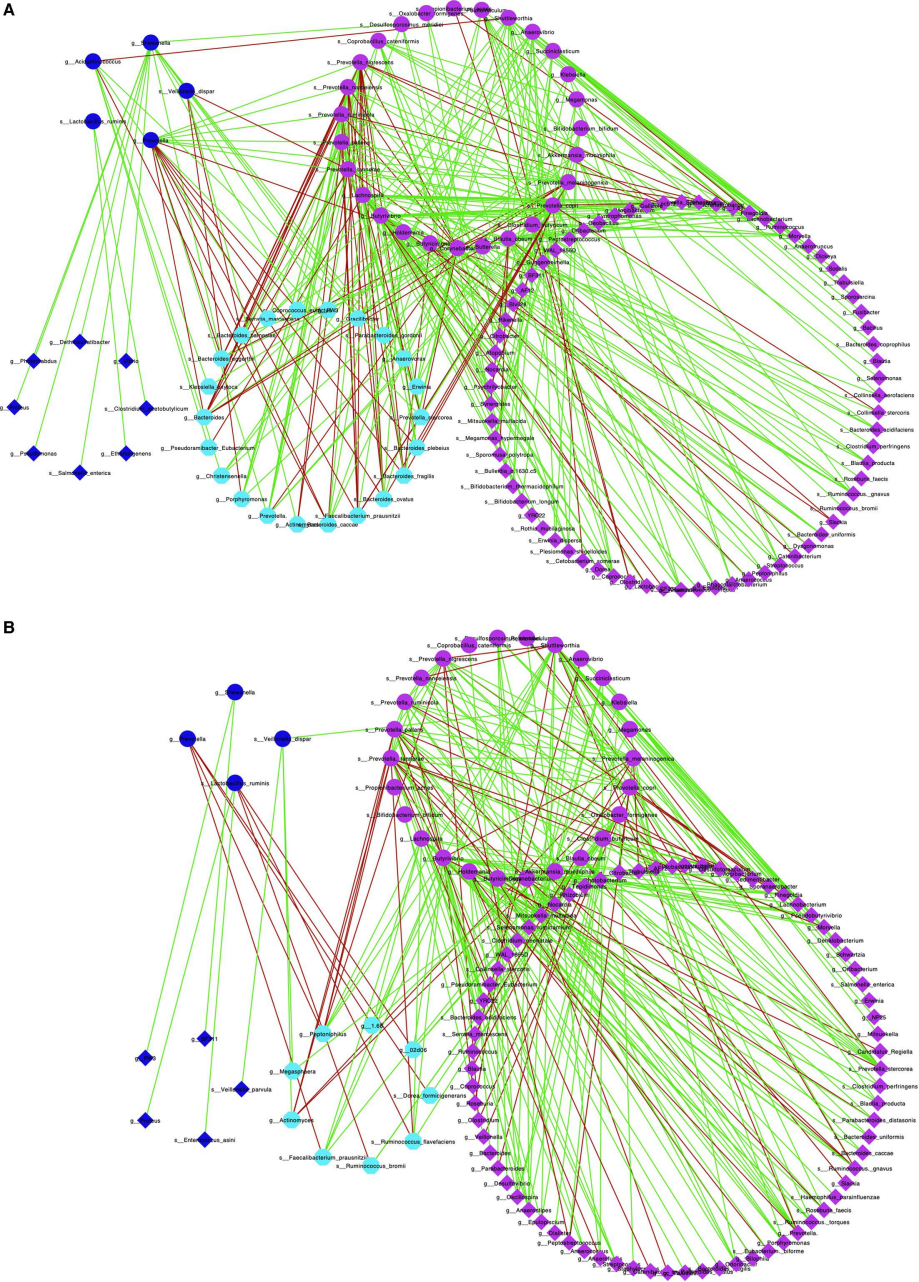
The first-order (nearest-neighbor) network of biomarker species in the HC and ASD groups. The FON graphs of biomarker spices and their neighbor species of the HC and ASD groups based on the SparCC algorithm and data set D2. (**A)** FON of HC cohort; (**B)** FON of ASD cohort; node in blue and round shape, the species with significantly decreased stiffness parameter; node in pink and round shape, species with significantly increased stiffness parameter; node in blue and diamond shape, the first-order neighbor species of biomarker species with significantly decreased stiffness parameter; node in pink and diamond shape, the first-order neighbor species of biomarker species with significantly increased stiffness parameter; node in cyan and hexagonal shape, the shared neighbor species of biomarker species; edge in green, positive correlations; edge in red, negative correlations.

The objective of constructing first-order neighbor (FON) network was to identify clusters of networks associated with biomarkers in the ASD gut microbiome. The FON biological maps of these biomarkers in HC and ASD cohorts of data set D2 were outlined, as illustrated in Fig. 3A and B, respectively. The analysis revealed that the ASD gut microbial biomarkers were organized into three clusters in both HC and ASD cohorts.

Cluster I (pink nodes in round shape) comprised biomarkers with significantly increased stiffness parameters and their neighboring species (pink nodes in diamond shape).

Cluster II (blue nodes in round shape) included biomarkers with significantly decreased stiffness parameters along with their neighbors (blue nodes in diamond shape).

Both clusters I and II were connected to a shared cluster III (cyan nodes in round shape), which plays the dual neighbor roles.

The relationships between microbial bacteria and their neighbors were quantified using the P/N ratio (the ratio of positive edges to negative edges) in the FON network. Table 3 presents the P/N ratios for the four clusters across each study data set. Key findings from the FON networks in HC and ASD cohorts include the following:

(i) The gut microbial biomarkers and their neighboring species formed distinct clusters characterized by positive correlations, indicating cooperative relationships between biomarkers and their neighboring species.

**TABLE 3:** The P/N ratios between the critical clusters of the FON network in the ASD and HC cohorts^*a*^

File	FON of HC cohort				FON of ASD cohort			
	Between cluster I & NN	Between cluster I and shared NN	Between cluster II and NN	Between cluster II and shared NN	Between cluster I and NN	Between cluster I and shared NN	Between cluster II and NN	Between cluster II and shared NN
D1	1.5	1.571	8.667	1.8	0.875	0.706	4.5	2.8
D2	5.32	1.571	–	1.4	3.65	2.625	–	0.286
D3	1.357	1.932	–	–	1	2.568	0	–
D4	0.1	11.5	–	9.5	2	2.111	4.333	26
D5	10	5.25	–	3	6.576	4.286	–	–
D6	0.607	1.263	2	1.333	1.882	0.5	5	1.222
D7	0.815	1.128	21	2.387	1.333	1.45	35	4.5
D8	0.756	0.8	1	2.667	0.975	0.812	1.6	1.643
**Mean**	**2.557**	**3.127**	**8.167**	**3.1557**	**2.2867**	**1.887**	**8.406**	**6.0756**
**Std. err.**	**3.4166**	**3.6606**	**9.207**	**2.867**	**1.956**	**1.281**	**13.17**	**9.87**

^*a*^ The symbol “-” indicates that there are only positive correlations and no negative correlations between the microbial sub-communities (clusters); cluster I is a sub-community of microbial bacteria with significantly increased network stiffness parameter; cluster II in term of microbial bacteria with significantly decreased network stiffness parameter; shared NN repents for nearest neighbors of both cluster I and cluster II; NN denotes the nearest neighbors of the cluster (either I or II); Mean denotes the mean value; Std.err signifies the standard error; *D*_*i*_ is the data set number (e.g., D1 for data set 1).

(ii) The correlation between microbial bacteria in cluster I (or cluster II) was positive (edge in green), while the correlation between primary cluster nodes (round nodes in pink or blue) in cluster I (or cluster II) and shared cluster nodes (nodes in cyan and hexagonal shape) was negative (edge in red). These results suggested that microbial bacteria of ASD gut microbiome in cluster I (or cluster II) form an “allies” sub-community in which microbial bacteria are competitive. The primary microbial bacteria in cluster I (or cluster II) and their shared neighbor species form an “foes” sub-community in which microbial species are cooperative.

(iii) The mean P/N ratio between the primary bacteria in cluster I and their neighbors (P/N(HC) = 2.557 or P/N(ASD) = 2.2867) was lower than the mean P/N ratio for cluster II and their neighbors (P/N(HC) = 8.167 or P/N(ASD) = 8.406) in both HC and ASD cohorts, as listed in Table 3. This indicates that biomarkers with significantly decreased network stiffness parameters are more likely to cooperate with their neighbors in both cohorts (P/N(HC) = 8.167 and P/N(ASD) = 8.406). Furthermore, the mean P/N(ASD) ratio between the primary bacteria of cluster I and shared cluster bacteria was 1.887, which was lower the mean P/N ratio between the primary bacteria of cluster II and shared cluster bacteria (P/N(ASD) = 6.0756) in the ASD cohort. No significant difference was observed between the clusters in the HC cohort (3.127 vs. 3.1557). These results suggest that the “allies” microbial bacteria are more inclined to cooperate with one another and play a role in promoting the dominance of biomarker bacteria within the gut microbiome of patients with ASD. Conversely, some microbial bacteria act as “foes” to the biomarkers (negatively correlated), potentially hindering the emergence of ASD gut microbiome biomarker bacteria through competitive interactions.

These findings imply that the co-occurrence patterns of disease-related bacteria in the ASD gut microbiome warrant further exploration.

## DISCUSSION

The objective of this study was to develop an algorithm known as the SNA method, which focuses on network stiffness as a topological property of complex networks to identify key microbial biomarkers in different states. By applying the SNA method to simulated and real data sets from the ASD gut microbiome, we demonstrated its efficacy in efficiently identifying more robust biomarkers and detecting biomarker subgroups often overlooked by traditional abundance-based methods.

The study comprised five fundamental steps: (i) quantifying differences in network stiffness parameters of species assemblages between ASD and HC cohorts, leading to the development of the SNA algorithm; (ii) identifying microbial biomarkers within the ASD gut microbiome; (iii) evaluating the performance of the SNA method in inferring RAs within networks; (iv) mining biomarker subgroups of the ASD gut microbiome; and (v) constructing and analyzing the FON network of microbial biomarkers in both ASD and HC cohorts.

In designing our research, we faced two options: quantifying differences in each of the eight datasets separately or pooling the data sets together for analysis. We opted for the first approach, measuring interindividual differences in network stiffness between the ASD and HC cohorts within the same case study. Over the past decade, findings from the Human Microbiome Project have shown that interindividual heterogeneity is inevitable (33–35), even when experimental designs are carefully controlled within the same case study. A common strategy for addressing interindividual variability is to analyze sufficiently larger sample sizes by pooling data sets. While this significantly increases sample size, there is no unified consensus on how to properly combine multiple data sets, as different case studies may introduce additional heterogeneity. This variability arises from differences in design, methodologies and operations among research groups and sequencing centers.

To navigate this challenge, we conducted SNA for each case study data set, generating distinct catalogs of microbial biomarkers (see Table S3 for the eight separate data sets). We further validated these categorizations by employing RF algorithms to reclassify randomly mixed validation samples into two distinct subtypes (ASD and HC cohorts), achieving classification precisions ranging from 82% to 95%.

The application of our stiffness network method not only facilitates the identification of microbial biomarkers for clinical diagnosis but also lays a foundation for further investigation into the etiology of ASD. Additionally, it supports the potential for automated diagnostics based on cost-effective amplicon sequencing and advancements in artificial intelligence technology.

We postulated that the RAs among microbial bacteria within sub-communities are unlikely to follow a monotonic pattern in the human gut microbiome of ASD patients. One plausible explanation is that the occurrence and progression of ASD are closely related to the selection of gut microbiome niches, which is influenced by host physiology and genomics, the severity of ASD symptoms, lifestyle factors, and dietary habits. The selection pressure on gut microbiome niches can be either favorable or unfavorable for certain microbial sub-communities, potentially increasing or decreasing the diversity of microbial species, as well as lowering or raising sub-community heterogeneity. The selection force of gut microbiome niches may be favorable or unfavorable for microbial bacteria of certain sub-communities, which may increase or decrease the diversity of microbial species and lower and raise the sub-community heterogeneity. The intuitive manifestation of the selection force is a redistribution of microbial abundance in space and time, and this redistribution is essentially “zero-sum”. Such redistributions underscore the complexity of restoring a dysfunctional microbiome to a balanced state. For instance, fecal microbiota transplantation has emerged as a potential treatment option for ASD patients, showing promise in alleviating some symptoms in affected children. However, due to the complexity, multi-dimension, and community heterogeneity of the ASD gut microbiome, there remains a lack of comprehensive safety evaluation strategies and reliable implementation plans for fecal microbiota transplantation.

## MATERIALS AND METHODS

### Study design and sample collection

**Fig 4:**
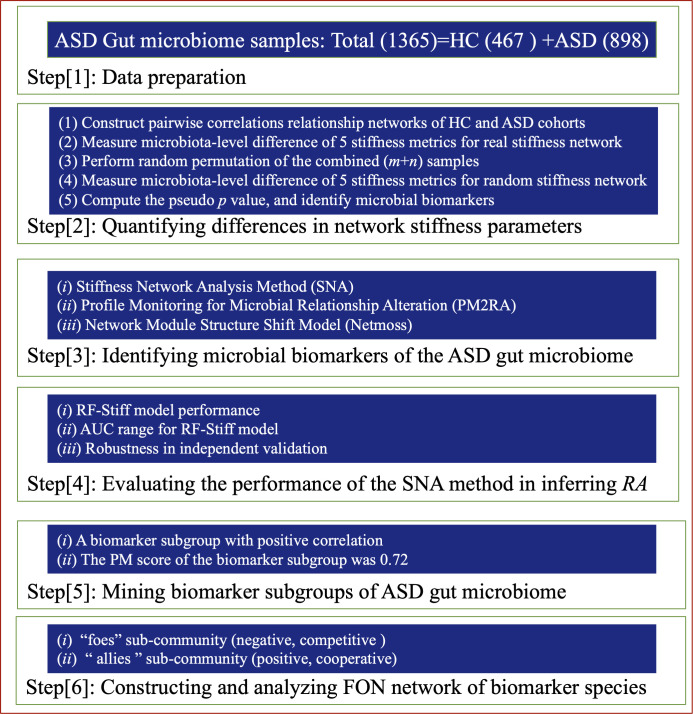
Study design for testing, measuring, and applying to the ASD gut microbiome samples.

This study analyzed 1,365 gut microbiome samples, comprising 898 from ASD patients and 467 from HC, collected from five countries: China, Ecuador, India, Italy, and the USA (see Table S1 for details). The 16S-rRNA sequencing data sets were retrieved from the NCBI database (https://www.ncbi.nlm.nih.gov/). Species classification was conducted using the 16S_Greengenes_k2db reference sequence database (March 25, 2020) with Kraken2 (version 2.1.2) and Bracken (version 2.6). In this study, the SNA method was developed for the first time, along with network module mining and FON network analysis, to thoroughly examine gut microbial biomarkers for ASD by reanalyzing the collected microbiome data sets. Fig. 4 outlines an overview of the study design, which consists of the following six steps (1–6).

### SNA: developing computational approaches

#### Force

Considering network stiffness as a topological property of complex networks allows computing (in a complex network) either the *forces* applied to network nodes, when the effect of forces is measurable, or the deformations (*displacements*) caused to network nodes by known forces, in accordance with the existing methodology in structural mechanics. Based on the analogy, which was previously described, we can assume the weights matrix *W*_*n* × *n*_ = [*w_ij_*] of a complex network as the stiffness matrix *K*_*n* × *n*_ = [*k_ij_*] of a structured framework.

Let us also consider a node-attribute (or activity) *X*, which measured at two different states such as *X* = {*x*(*t_a_*) = HC and *x*(*t_b_*) = ASD}. In this report, we assume the change *x*(*t_b_*) *− x*(*t_a_*) observed for this attribute is the displacements [*d* = *d*(*t_b_*) − *d*(*t_a_*) = *x*(*t_b_*) − *x*(*t_a_*)] caused by an external (or internal) force *f*. Then, we can compute the force applied to the set of network nodes *V_G_*:


(1) $$f^{n\times 1}=K^{n\times n}d_{observed}^{n\times 1}=Wd_{observed}=W{\left[d_{i}\left(t_{b}\right)-d_{i}\left(t_{a}\right)\right]}^{n\times 1}\;\;\;i=1,2,\ldots ,n,$$

where *n* is the number of network nodes. In structured frameworks, the forces vector *f*_*n* × 1_ = [*F_i_*] is measured in force units, whereas in complex networks, forces are measured in *wx* units. To guarantee symmetry, we assume that the changes (*x*(*t_b_*) − *x*(*t_a_*)) of the attribute, which is observed from the condition HC to ASD, are equal to those from the condition ASD to HC.

#### Displacement

In contrast, by considering that a known force *f* is applied to the set of network nodes *V_G_*, we can compute the effect of this force (i.e., the displacements vector $d_{n\times 1}={\left(d_{1},d_{2},\cdots {,d}_{n}\right)}^{T}$on *V_G_* by solving the linear system (1) and obtain


(2) $$d^{n\times 1}={\left(W_{n\times n}\right)}^{-1}f_{observed}^{n\times 1}$$

where (*W_n × n_*) ^−1^ is the inverse of the weight matrix *W_n × n_* = [*w_ij_*] and the weight matrix *W_n×n_* is non-singular. In structured frameworks, the displacement vector *d* expresses the length of displacements along the direction of the external force *f* and is measured in metric units. In complex networks, *d* expresses the effect of *f* on the network nodes, and it is measured in *F/w* (force/weights) units.

According to relation (1), when *d* is a unit vector and the adjacency matrix *A_n×n_* = [*a_ij_*] is the stiffness matrix (*K* = *A*), then the forces vector *f* equals to the network degrees and *f* ≡*k* = (*k*_1_, *k*_2_,…, *k*_n_). This observation implies that when the vector of network degrees is applied as force to an unweighted network (i.e., every node is subjected to a force equal to its degree), it causes unit effects (displacements).

Furthermore, it can be also observed that when a single node is subjected to a force equal to its degree, the displacements caused to the other network nodes is equal to the degree of the nodes while the force is applied. In other words, when the vector of network strengths is applied as force *f* ≡ *k* = (*k*_1_, *k*_2_,…, *k*_n_) to a weighted network, it causes unit effects (displacements) to network nodes.

#### Stiffness scale

In structural engineering, network stiffness is a concept linking the vectors of forces (*f*) and displacements (*d*) in a cause-effect context. This is because the stiffness matrix *K* is the tensor expressing a pairwise relation in the form *K* = *R*(*f*, *d*). Within this framework, by assuming that a complex network behaves similarly to a structured framework, we can develop a complex network and a pairwise property between any pair of node attributes ***x*** and ***y***, for which a cause-effect pattern of the form *y* = *R*(*x*) will exist (*x* = cause, *y* = effect). Within a structured framework such as the context, this is possible by considering that one network attribute acts as the vector of forces (e.g., *y* = *f*), while the second acts as the vector of displacements (*x* = *d*).

Let us consider a complex network *G* (*V*, *E*) with stiffness *K,* and a pair of node variables (*x*, *y*) have the length |*V*| = *n*. According to relation (1), node variables *x*, *y* can attain a cause-effect relation within the context of network stiffness as follows:


(3) $$f=Kd\Leftrightarrow x=Ky\Rightarrow y=K^{-1}x.$$

If we assume that the stiffness matrix is the connectivity matrix (*K≡W*), then relation (3) cannot be satisfied for the node variables *x*, *y*. Therefore, we need to define the stiffness matrix in terms of *W*, namely, *K* = *f*(*W*). To do so, let us consider a vector ***s*** = [*s*_1_, *s*_2_,..., *s*_n_]′ and the diagonal function diag(.), as follows:


(4) $$diag\left(s\right)=diag\left(s_{1},s_{2},\cdots s_{n}\right)=S_{d}\left(s\right)=\left(\begin{array}{ccc} s_{1} & & \\ & \begin{array}{cc} s_{2} & \\ & \ddots \end{array} & \\ & & s_{n} \end{array}\right)=\left\{ \begin{array}{l} \begin{array}{cc} s_{1} & i=j \end{array}\\ \begin{array}{cc} o & \;otherwise \end{array} \end{array}\right..$$

Then, we seek a matrix ***S****_d_* (*s*) that produces the stiffness matrix *K*, according to references (36, 37)


(5) $$K=S_{d}\left(s\right).W.$$

Based on relations (3) to (5), we obtain:


(6) $$x=Ky⟺f=Kd=S_{d}\left(s\right)Wd=\left(\begin{array}{ccc} s_{1}.w_{11} & \cdots & s_{1}.w_{1n}\\ \vdots & \ddots & \vdots \\ s_{n}.w_{n1} & \cdots & s_{n}.w_{nn} \end{array}\right)d,$$

where *x* = [*x_ij_*], *f* = [*F_ij_*], *y* = [*y_ij_*], *d* = [*d_ij_*], *W_n×n_* = [*w_ij_*] is the connectivity (weights) matrix of network *G*, and we have


(7) $$s=\left[\frac{x_{1}}{\sum_{j=1}^{n}w_{1j}y_{j}},\frac{x_{2}}{\sum_{j=1}^{n}w_{2j}y_{j}},\cdots ,\frac{x_{n}}{\sum_{j=1}^{n}w_{nj}y_{j}}\right].$$

#### Impact

Given an activity (node-attribute) *X*, which for the nodes *i* and *j* takes values *X* = *x_i_* and *X* = *x_j_*, respectively, the correlation matrix has been defined by the permanent perturbations *dx_i_* on the value *x_i_* and *dx_j_* on the value *x_j_*, according to the formula:


(8) $$G_{ij}\left(x\right)=\left|\frac{dx_{i}/x_{i}}{dx_{j}/x_{j}}\right|.$$

This correlation matrix [Eqn. 8], which is used to capture the influence of node *j* on *i*.

Based on this matrix, Barzel et al. (38) defined the impact of node *i*, according to the formula:


(9) $$I_{i}\left(X\right)=\sum_{j=1}^{n}A_{ij}G_{ij}^{T},$$

where *A_ij_* is the adjacency matrix of the network. Equation 9 captures the average response of the node’s (*i*) neighborhood to the perturbation of *i.*

#### Stability

Barzel et al. (38) also defined the stability of node *i*, according to the following formula:


(10) $$S_{i}\left(X\right)=\frac{1}{\sum_{j=1}^{n}A_{ij}G_{ij}}.$$

Equation 10 is different from Equation 9 in that node resilience is defined by the *G_ij_* matrix instead of by the transposed (*G_ij_^T^*). Node stability captures the inverse response of node *i* to individual perturbations of its nearest neighbors, which ranges from 0 to 1. If the node stability is close to 0, it indicates that the community assembly is susceptible to disturbance. If the node stability is close to 1, it indicates that the community assembly is relatively stable.

### Calculation steps for the permutated networks and real networks

#### Step [1]

The pairwise correlation relationship networks of HC (m samples) and ASD (n samples) cohorts are constructed for each study data set based on the weighted correlation network analysis and the relative abundance of samples.

#### Step [2]

Changes in network stiffness parameters are investigated by the SNA method.

#### Step [3]

Pool the HC and ASD samples of each study data set together, and perform random permutation of the combined (*m + n*) samples. The first *m* samples are taken as the permutated HC cohort, and the remaining *n* samples are taken as the permutated ASD cohort. We conduct 1,000 random permutations for each study data set.

#### Step [4]

The network stiffness parameters of each pairwise permutated HC and ASD cohorts are calculated using the algorithm in Step [1] and Step [2].

#### Step [5]

Compute the pseudo-*P* value. The pseudo-*P* value can be defined as the ratio of the permutations with *R_i_* < *R* (*R_i_* ≥ *R*) (*R*: the real value of network stiffness parameter (*s*, *I*, *S*) and *R_i_*: the random value of network stiffness parameters) among 1,000 permutations. Assume *N_1_* and *N_2_* are the numbers of times satisfying *R_i_* < *R* and *R_i_* ≥ *R* in 1,000 permutation test, respectively. Then, *P*_1_ = *N*_1_/1,000, *P*_2_ = *N*_2_/1,000. Obviously, if FDR < 0.05, the species is significantly higher than permutated simulation, which is defined as significantly increased species. If FDR < 0.05, the species is significantly lower than permutated simulation, which is defined as significantly decreased species.

## Data Availability

All analyzed data sets are available in the public domain. The original contributions presented in the study are included in the article and Supplemental material; further inquiries can be directed to the corresponding authors.

## SUPPLEMENTAL MATERIAL

Supplemental figures spectrum.01078-24-s0001.docx Fig. S1 and S2.

Supplemental tables spectrum.01078-24-s0002.docx Table S1 to S5.

ASM does not own the copyrights to Supplemental Material that may be linked to, or accessed through, an article. The authors have granted ASM a non-exclusive, world-wide license to publish the Supplemental Material files. Please contact the corresponding author directly for reuse.
