# Supplementary material for: Resilience of human gut microbiomes in autism spectrum disorder: measured using stiffness network analysis
Source: Microbiol Spectr. 2025 Feb 4;13(3):e01078-24. doi: 10.1128/spectrum.01078-24 (PMC11878074; doi:10.1128/spectrum.01078-24)
Supplement: Supplemental figures — Fig. S1 and S2. [file spectrum.01078-24-s0001.docx]

**Figure S1.** The relationship alternates (RA) difference in the "allies" biomarker subgroup consisting of four biomarker bacteria in the gut microbiome of patients with ASD

**Note:** the distributions of Hotelling’s *T^2^* statistics for four biomarker bacteria shown in the ASD and HC cohorts, the vertical coordinate is the frequency (%). The overlapping area (PM score) represents the relationship alterations of the "allies" biomarker subgroup in the ASD gut microbiome between two different states (PM score=0.72).

**Figure S2.** Relative abundance of four biomarker bacteria in ASD and HC cohorts.

Note: The horizontal coordinate represents the relative abundance of species with log-transformation and the vertical coordinate is the frequency (%).
