# Supplementary material for: Resilience of human gut microbiomes in autism spectrum disorder: measured using stiffness network analysis
Source: Microbiol Spectr. 2025 Feb 4;13(3):e01078-24. doi: 10.1128/spectrum.01078-24 (PMC11878074; doi:10.1128/spectrum.01078-24)
Supplement: Supplemental tables — Table S1 to S5. [file spectrum.01078-24-s0002.docx]

**Online Supplementary Information for** Chen *et al.* (2024) Resilience of Human Gut Microbiomes in Autism Spectrum Disorder: Measured Using Stiffness Network Analysis

**Table S1.** Summary of human gut microbiome samples for patients with autism, including 898 ASD samples and 467 health control samples

| **Dataset No.** | **Dataset ID** | **Total Sample Size** | **Cohort** | **Sample Size** | **Mean of reads** | **Country & Year** | **Reference** |
| --- | --- | --- | --- | --- | --- | --- | --- |
| D1 | PRJEB27306 | 58 | HC | 27 | 19200.548 | Ecuador  2019 | Zurita et al. (2020). Gut Microbes, 11(3):453-464. |
|  |  |  | ASD | 31 | 18704.481 |  |  |
| D2 | PRJNA355023 | 50 | HC | 30 | 659867.8 | India  2017 | Pulikkan et al. (2018). Microbial Ecology, 76(4):1102-1114. |
|  |  |  | ASD | 20 | 782236.467 |  |  |
| D3 | PRJNA453840 | 346 | HC | 173 | 74279.572 | China  2021 | Liu et al. (2021).  Gut Microbes, 13(1):1-16 |
|  |  |  | ASD | 173 | 77450.139 |  |  |
| D4 | PRJNA589343 | 127 | HC | 77 | 85627.24 | China  2020 | Ding et al. (2020).  Journal of Psychiatric Research, 129:149-159. |
|  |  |  | ASD | 50 | 98471.286 |  |  |
| D5 | PRJNA642975 | 86 | HC | 44 | 24791.714 | China  2020 | Cao et al. (2021). Frontiers in Neuroscience, 15:635925. |
|  |  |  | ASD | 42 | 25088.295 |  |  |
| D6 | PRJNA644763 | 123 | HC | 76 | 52740.021 | China  2020 | Chen et al. (2020). mSphere, 5(6): e01044-20. |
|  |  |  | ASD | 47 | 53740.842 |  |  |
| D7 | PRJNA687773 | 83 | HC | 39 | 124604.773 | China  2021 | Huang et al. (2021). Frontiers in Psychiatry,12:682454. |
|  |  |  | ASD | 44 | 126762.103 |  |  |
| D8 | PRJNA769228 | 198 | HC | 138 | 48360.65 | China  2021 | Chen et al. (2021). Frontiers in Psychiatry, 12:789864. |
|  |  |  | ASD | 60 | 43381.986 |  |  |
| **Total** | | **1071** | **HC：467**  **ASD：604** | | | | |
| DS1 | PRJEB15418 | 71 | ASD | 71 | 34871.141 | Italy  2020 | Strati et al. (2017). Microbiome, 5(1):24. |
| DS2 | PRJEB29421 | 25 | ASD | 25 | 55886.6 | Italy  2018 | Coretti et al. (2018). Frontiers in Microbiology, 9:3146. |
| DS3 | PRJEB42687 | 78 | ASD | 78 | 96467.74 | USA  2021 | Fouquier et al. (2021). mSystems, 6(2): e00848-20. |
| DS4 | PRJNA615774 | 120 | ASD | 120 | 56288.742 | China  2020 | Ho et al. (2020).  Gut Pathogens, 11:855. |
| **Total** | | **294** |  | **ASD：294** |  |  |  |
| D9 |  | 468 | HC | 234 |  |  |  |
|  |  |  | ASD | 234 |  |  |  |

Dataset D9 consisted of 234 HC samples and 234 ASD samples, of which 234 ASD samples were randomly selected from 294 samples (DS1, DS2, DS3 and DS4), and 234 HC samples were randomly selected from HC cohort of 8 datasets.

**References**

Cao, X., Liu, K., Liu, J., Liu, Y. W., Xu, L., Wang, H., Zhu, Y., Wang, P., Li, Z., Wen, J., Shen, C., Li, M., Nie, Z., Kong, X. J. (2021). Dysbiotic Gut Microbiota and Dysregulation of Cytokine Profile in Children and Teens with Autism Spectrum Disorder. Frontiers in Neuroscience, 15, 635925.

Chen, Y., Fang, H., Li, C., Wu, G., Xu, T., Yang, X., Zhao, L., Ke, X., & Zhang, C. (2020). Gut Bacteria Shared by Children and Their Mothers Associate with Developmental Level and Social Deficits in Autism Spectrum Disorder. mSphere, 5(6), e01044-20.

Coretti, L., Paparo, L., Riccio, M. P., Amato, F., Cuomo, M., Natale, A., Borrelli, L., Corrado, G., Comegna, M., Buommino, E., Castaldo, G., Bravaccio, C., Chiariotti, L., Berni Canani, R., & Lembo, F. (2018). Gut Microbiota Features in Young Children with Autism Spectrum Disorders. Frontiers in Microbiology, 9, 3146.

Chen, Z., Shi, K., Liu, X., Dai, Y., Liu, Y., Zhang, L., Du, X., Zhu, T., Yu, J., Fang, S., & Li, F. (2021). Gut Microbial Profile Is Associated with the Severity of Social Impairment and IQ Performance in Children with Autism Spectrum Disorder. Frontiers in Psychiatry, 12, 789864.

Ding, X., Xu, Y., Zhang, X., Zhang, L., Duan, G., Song, C., Li, Z., Yang, Y., Wang, Y., Wang, X., & Zhu, C. (2020). Gut microbiota changes in patients with autism spectrum disorders. Journal of Psychiatric Research,129, 149-159.

Fouquier, J., Moreno Huizar, N., Donnelly, J., Glickman, C., Kang, D. W., Maldonado, J., Jones, R. A., Johnson, K., Adams, J. B., Krajmalnik-Brown, R., & Lozupone, C. (2021). The Gut Microbiome in Autism: Study-Site Effects and Longitudinal Analysis of Behavior Change. mSystems, 6(2), e00848-20.

Huang, M., Liu, J., Liu, K., Chen, J., Wei, Z., Feng, Z., Wu, Y., Fong, M., Tian, R., Wang, B., Budjan, C., Zhuang, P., Wan, G., & Kong, X. J. (2021). Microbiome-Specific Statistical Modeling Identifies Interplay Between Gastrointestinal Microbiome and Neurobehavioral Outcomes in Patients with Autism: A Case Control Study. Frontiers in Psychiatry, 12, 682454.

Ho, L. K. H., Tong, V. J. W., Syn, N., Nagarajan, N., Tham, E. H., Tay, S. K., Shorey, S., Tambyah, P. A., & Law, E. C. N. (2020). Gut microbiota changes in children with autism spectrum disorder: a systematic review. Gut Pathogens, 12, 6.

Liu, Z., Mao, X., Dan, Z., Pei, Y., Xu, R., Guo, M., Liu, K., Zhang, F., Chen, J., Su, C., Zhuang, Y., Tang, J., Xia, Y., Qin, L., Hu, Z., & Liu, X. (2021). Gene variations in autism spectrum disorder are associated with alteration of gut microbiota, metabolites and cytokines. Gut Microbes, 13(1), 1-16.

Pulikkan, J., Maji, A., Dhakan, D. B., Saxena, R., Mohan, B., Anto, M. M., Agarwal, N., Grace, T., & Sharma, V. K. (2018). Gut Microbial Dysbiosis in Indian Children with Autism Spectrum Disorders. Microbial Ecology, 76(4), 1102-1114.

Strati F, Cavalieri D, Albanese D, et al. (2017) New evidences on the altered gut microbiota in autism spectrum disorders. Microbiome, 5(1):24.

Zurita, M. F., Cárdenas, P. A., Sandoval, M. E., Peña, M. C., Fornasini, M., Flores, N., Monaco, M. H., Berding, K., Donovan, S. M., Kuntz, T., Gilbert, J. A., Baldeón, M. E. (2020). Analysis of gut microbiome, nutrition and immune status in autism spectrum disorder: a case-control study in Ecuador. Gut Microbes, 11(3), 453-464.

**Table S2.** Basic network properties and the P/N (positive to negative links) ratios of the ASD gut microbial networks for each cohort of case study dataset

| **Dataset** | **Cohorts** | **Num. of Nodes** | **Num. of Edges** | **Avg. Local Cluster Coefficient** | **Average Path Length** | **Network Density** | **Network Modularity** | **Num. of Communities** | **Positive Links (+)** | **Negative Links (-)** | **P/N (+/-) Ratio** |
| --- | --- | --- | --- | --- | --- | --- | --- | --- | --- | --- | --- |
| D1 | HC | 549 | 98240 | 0.875 | 1.156 | 0.653 | 0.004 | 3 | 43758 | 54482 | 0.803 |
|  | ASD | 549 | 99329 | 0.859 | 1.152 | 0.66 | 0.004 | 3 | 43651 | 55678 | 0.784 |
| D2 | HC | 552 | 62476 | 0.783 | 1.565 | 0.411 | 0.028 | 4 | 58588 | 3888 | 15.069 |
|  | ASD | 534 | 36152 | 0.673 | 3.096 | 0.254 | 0.057 | 10 | 35959 | 193 | 186.316 |
| D3 | HC | 498 | 50893 | 0.823 | 2.515 | 0.411 | 0.058 | 4 | 40555 | 10338 | 3.923 |
|  | ASD | 497 | 45930 | 0.81 | 2.457 | 0.373 | 0.077 | 8 | 40241 | 5689 | 7.073 |
| D4 | HC | 547 | 82481 | 0.857 | 2.008 | 0.552 | 0.019 | 3 | 67890 | 14591 | 4.653 |
|  | ASD | 537 | 74688 | 0.815 | 1.281 | 0.519 | 0.016 | 2 | 44012 | 30676 | 1.435 |
| D5 | HC | 551 | 97568 | 0.881 | 1.123 | 0.644 | 0.014 | 2 | 49917 | 47651 | 1.048 |
|  | ASD | 525 | 56373 | 0.821 | 2.68 | 0.41 | 0.049 | 9 | 51893 | 4480 | 11.583 |
| D6 | HC | 532 | 84701 | 0.839 | 1.226 | 0.6 | 0.006 | 3 | 36737 | 47964 | 0.766 |
|  | ASD | 532 | 78548 | 0.822 | 1.291 | 0.556 | 0.008 | 4 | 39989 | 38559 | 1.037 |
| D7 | HC | 535 | 72855 | 0.82 | 1.274 | 0.51 | 0.016 | 4 | 41331 | 31524 | 1.311 |
|  | ASD | 550 | 64788 | 0.768 | 1.527 | 0.429 | 0.021 | 2 | 43260 | 21528 | 2.009 |
| D8 | HC | 526 | 70650 | 0.808 | 1.362 | 0.512 | 0.01 | 4 | 34683 | 35967 | 0.964 |
|  | ASD | 509 | 63641 | 0.805 | 1.314 | 0.492 | 0.018 | 2 | 33394 | 30247 | 1.104 |
| **Mean** | **HC** | **536** | **77483** | **0.836** | **1.53** | **0.537** | **0.019** | **3.375** | **46682** | **30800.7** | **3.567** |
|  | **ASD** | **529** | **64931** | **0.797** | **1.850** | **0.461** | **0.031** | **5** | **41549.9** | **23381** | **26.417** |

**Table S3.** Microbial biomarkers of the ASD gut microbiome using SNA Method

| **Species** | **Phylum** | **Category** | **Refference** |
| --- | --- | --- | --- |
| *Lachnospira* | *Firmicutes* | Pathogenesis | Grimaldi et al. (2018). Microbiome, 6:133.  Zou et al. (2020). Autism Research, 13:1614-1625.  Liu et al. (2022). Experimental Dermatology, 31(2):242-247. |
| *Parabacteroides distasonis* | *Bacteroidetes* | Pathogenesis | Finegold et al. (2010). Anaerobe, 16:444-453.  Buffington et al. (2016). Cell, 165:1762-1775.  Coretti et al. (2017). Scientific Reports, 7:45356.  Golubeva et al. (2017). EBioMedicine, 24:166-178. |
| *Faecalibacterium prausnitzii* | *Firmicutes* | Opportunistic pathogen | Coretti et al. (2018). Frontiers in Microbiology, 9:3146.  Ding et al. (2020). Journal of Psychiatric Research, 129:149-159.  Wan et al. (2021). Gut, 71:910-918. |
| *Ruminococcus gnavus* | *Firmicutes* | Pathogenesis | Finegold et al. (2002). Clinical Infectious Diseases, 35:6-16.  Finegold et al. (2010). Anaerobe, 16:444-453.  Coretti et al. (2017). Scientific Reports, 7:45356.  Dan et al. (2020). Gut Microbes, 11:1246-1267. |
| *Phascolarcto bacterium* | *Firmicutes* | Unclassified | Rachek et al. (2021). Microbiology Resource Announcements, 10(4): e01054-20. |
| *Odoribacter* | *Bacteroidetes* | Pathogenesis | Golubeva et al. (2017). EBioMedicine, 24:166-178.  Zhang et al. (2018). Scientific Reports, 8:13981.  Altimiras et al. (2021). Frontiers in Neuroscience, 15:653120. |
| *Dorea* | *Firmicutes* | Pathogenesis | Luna et al. (2017). Cellular and Molecular Gastroenterology and Hepatology, 3:218-230.  Ding et al. (2020). Journal of Psychiatric Research, 129:149-159. |
| *Dialister* | *Firmicutes* | Beneficial bacteria | Finegold et al. (2010). Anaerobe, 16:444-453.  Zou et al. (2020). Autism Research, 13:1614-1625.  Dan et al. (2020). Gut Microbes, 11:1246-1267.  Wan et al. (2021). Gut, 71:910-918. |
| *Coprococcus* | *Firmicutes* | Pathogenesis | Tabouy et al. (2018). Brain Behavior and Immunity, 73:310-319.  Cao et al. (2021). Frontiers in Neuroscience, 15:635925. |
| *Veillonella dispar* | *Firmicutes* | Pathogenesis | Zhang et al. (2018). Scientific Reports, 8:13981.  Tabouy et al. (2018). Brain Behavior and Immunity, 73:310-319.  Cobo et al. (2020). Anaerobe, 66:102285. |
| *Prevotella copri* | *Bacteroidetes* | Unclassed | Tett et al. (2019). Cell host & microbe, 26(5):666-679 |
| *Roseburia faecis* | *Firmicutes* | Unclassified | Tamanai-Shacoori et al. (2017). Future Microbiology, 12:157-170. |
| *Bacteroides ovatus* | *Bacteroidetes* | Unclassified | Coyne et al. (2019). Journal of Clinical Nursing, 28(21-22):4062-4076. |
| *Oscillospira* | *Firmicutes* | Pathogenesis | Coretti et al. (2017). Scientific Reports, 7:45356.  Coretti et al. (2018). Frontiers in Microbiology, 9:3146.  Altimiras et al. (2021). Frontiers in Neuroscience, 15:653120. |
| *Sutterella* | *Proteobacteria* | Pathogenesis | Williams et al. (2011). PloS one, 6: e24585.  Luna et al. (2017). Cellular and Molecular Gastroenterology and Hepatology, 3:218-230.  Coretti et al. (2017). Scientific Reports, 7:45356.  Zhang et al. (2018). Scientific Reports, 8:13981.  Altimiras et al. (2021). Frontiers in Neuroscience, 15:653120. |
| *Bacteroides plebeius* | *Bacteroidetes* | Unclassified | Pei et al. (2022). Journal of Cellular and Molecular Medicine, 26(24):6066-6078. |
| *Bacteroides caccae* | *Bacteroidetes* | Opportunistic pathogen | Finegold et al. (2010). Anaerobe, 16: 444-453.  Hsiao et al. (2013). Cell, 155:1451-1463.  Buffington et al. (2016). Cell, 165:1762-1775.  Coretti et al. (2017). Scientific Reports, 7:45356.  Golubeva et al. (2017). EBioMedicine, 24:166-178.  Tabouy et al. (2018). Brain Behavior and Immunity, 73:310-319.  Zou et al. (2020). Autism Research, 13:1614-1625.  Ding et al. (2020). Journal of Psychiatric Research, 129:149-159.  Altimiras et al. (2021). Frontiers in Neuroscience, 15:653120. |

**Table S4.** Microbial biomarkers of the ASD gut microbiome using PM2RA method (*FDR*<0.05 and *PM score* >0.6)

| **Species** | **Phylum** | **Category** | **Refference** |
| --- | --- | --- | --- |
| *Bacteroides uniformis* | *Bacteroides* | Pathogenesis | Buffington et al. (2016). Cell, 165:1762-1775. |
| *Bilophila* | *Proteobacteria* | Pathogenesis | Coretti et al. (2017). Scientific Reports, 7:45356.  Golubeva et al. (2017). EBioMedicine, 24:166-178. |
| *Prevotella.* | *Bacteroides* | Pathogenesis | Zou et al. (2020). Autism Research, 13:1614-1625. |
| *Bacteroides plebeius* | *Bacteroidetes* | Beneficial bacteri | Pei et al. (2022). Journal of Cellular and Molecular Medicine, 26(24):6066-6078. |
| *Sutterella* | *Proteobacteria* | Pathogenesis | Williams et al. (2011). PloS One, 6:e24585.  Coretti et al. (2017). Scientific Reports, 7:45356.  Luna et al. (2017). Cellular and Molecular Gastroenterology and Hepatology, 3:218-230.  Zhang et al. (2018). Scientific Reports, 8:13981.  Altimiras et al. (2021). Frontiers in Neuroscience, 15:653120. |
| *Lachnospira* | *Firmicutes* | Pathogenesis | Hsiao et al. (2013). Cell, 155:1451-1463.  Grimaldi et al. (2018). Microbiome, 6:133.  Zou et al. (2020). Autism Research, 13:1614-1625.  Liu et al. (2022). Experimental Dermatology, 31(2):242-247. |
| *Prevotella copri* | *Bacteroidetes* | Unclassed | Tett et al. (2019). Cell host & microbe, 26(5):666-679 |

**Table S5.** Microbial biomarkers of the ASD gut microbiome using Netmoss method (*Netmoss*>0.5)

| **Species** | **Phylum** | **Category** | **Refference** |
| --- | --- | --- | --- |
| *Roseburia faecis* | *Firmicutes* | Unclassified | Tamanai-Shacoori et al. (2017). Future Microbiology, 12:157-170. |
| *Bacteroides ovatus* | *Bacteroidetes* | Unclassified | Faith et al. (2021).  American Journal of Pathology,  191(4):704-719. |
| *Bacteroides caccae* | *Bacteroidetes* | Unclassified | Cheng et al. (2019).  Clinical Laboratory, 65(12). |
| *Bacteroides acidifaciens* | *Bacteroidetes* | Pathogenesis | Wang et al. (2022).  Gut Microbes, 14(1):2027853. |
| *Veillonella* | *Firmicutes* | Pathogenesis | Wicaksono et al. (2020). Applied and Environmental Microbiology, 86(20): e01255-20. |
| *Phascolarcto*  *bacterium* | *Firmicutes* | Unclassified | Wu et al. (2017).  Experimental and Therapeutic Medicine, 14(4):3122-3126. |
| *Odoribacter* | *Bacteroidetes* | Pathogenesis | Cleynen et al. (2016).  Lancet, 387(10014):156-167. |
| *Collinsella* | *Actinobacteria* | Pathogenesis | Gomez-Arango et al. (2018). Gut Microbes, 9(3):189-201. |
| *Blautia* | *Firmicutes* | Pathogenesis | Liu et al. (2021).  Gut Microbes, 13(1):1-21. |
| *Bilophila* | *Proteobacteria* | Pathogenesis | Coretti et al. (2017). Scientific Reports, 7:45356.  Golubeva et al. (2017). EBioMedicine, 24:166-178. |
| *Bacteroides* | *Bacteroidetes* | Pathogenesis | Zafar et al. (2021).  Gut Microbes, 13(1):1-20. |
| *Anaerostipes* | *Firmicutes* | Opportunistic pathogen | Bui et al. (2021). Nature Communication, 12(1):4798. |
| *Bacteroides plebeius* | *Bacteroidetes* | Unclassified | Pei et al. (2022). Journal of Cellular and Molecular Medicine, 26(24):6066-6078. |
| *Sutterella* | *Proteobacteria* | Pathogenesis | Williams et al. (2011). PloS One, 6: e24585.  Coretti et al. (2017). Scientific Reports, 7:45356.  Luna et al. (2017). Cellular and Molecular Gastroenterology and Hepatology, 3:218-230.  Zhang et al. (2018). Scientific Reports, 8:13981.  Altimiras et al. (2021). Frontiers in Neuroscience, 15:653120. |
| *Lachnospira* | *Firmicutes* | Pathogenesis | Hsiao et al. (2013). Cell, 155:1451-1463.  Grimaldi et al. (2018). Microbiome, 6:133.  Zou et al. (2020). Autism Research, 13:1614-1625.  Liu et al. (2022). Experimental Dermatology, 31(2):242-247. |
| *Prevotella copri* | *Bacteroidetes* | Unclassed | Tett et al. (2019). Cell host & microbe, 26(5):666-679. |
